# Physical activity during recess among 13–14 year old Mexican girls

**DOI:** 10.1186/s12887-015-0329-4

**Published:** 2015-03-05

**Authors:** Catalina Medina, Simon Barquera, Peter T Katzmarzyk, Ian Janssen

**Affiliations:** School of Kinesiology and Health Studies, Queen’s University, Kingston, ON Canada; Mexican National Institute of Public Health, Av. Universidad 655, Col. Sta. María Ahuacatitlán, 62100 Cuernavaca, Morelos Mexico; Pennington Biomedical Research Center, Baton Rouge, LA USA

**Keywords:** Adolescents, Physical activity, Secondary schools, Mexico

## Abstract

**Background:**

Physical activity patterns during recess have been poorly described among adolescents. Physical activity levels could be captured the most accurately using a combination of instruments. The purposes of this study were to describe the physical activity patterns during school recess in a sample of 13–14 year old Mexican girls, to examine differences in these patters as assessed using three physical activity measurement instruments and to understand the influence of body weight status on the observed associations.

**Methods:**

The study population included seventy-two female adolescents from a private school in Mexico City. Three different instruments were used to monitor physical activity patterns during the recess break including an accelerometer, direct observation (SOFIT instrument), and a physical activity recall. Descriptive analyses were used to characterize physical activity patterns, and one-way ANOVA was used to examine differences across physical activity instruments.

**Results:**

Based on the accelerometer data, more than 90% of the recess period was spent at a light or sedentary intensity. Based on SOFIT and recall, the two most frequent activities were standing and walking. There was a significant difference (p < 0.05) between the three instruments for time spent in all physical activity intensities.

**Conclusion:**

The large amount of time spent in light and sedentary intensity activities during recess indicates the necessity to intervene upon this opportunity for adolescents to engage in more vigorous forms of physical activity.

## Background

Regular physical activity during childhood and adolescence is associated with a lower risk of obesity, insulin resistance, mental health problems, and improved academic performance [1,2]. Unfortunately, meeting physical activity guidelines is a public health challenge, specifically in adolescents where physical activity levels tend to decline markedly [3-5]. In Mexico, 22.7% of 15–19 year old adolescents are not physically active (achieving at least 60 minutes of moderate-to-vigorous physical activity daily) [6], while 13% of adolescents are obese [6]. Therefore, a high priority should be given to implementing strategies to increase physical activity in this age group.

On weekdays, children and adolescents spent approximately 40% of their waking time at school, and the school environment can influence their physical activity behaviors [7-9]. Although there are no formal guidelines for physical activity levels during the school recess period, Ridgers et al. [8] suggested that between 5 and 40% of the daily volume of physical activity needed to meet public health recommendations can be accumulated during recess in developed countries such as Portugal, the United States, and the United Kingdom. In Mexico, a developing country, most secondary schools have a mandatory 30-minute recess/lunch period in which students have the opportunity to eat and be physically active.

There is a dearth of information about physical activity levels during the recess period in adolescents. The available research indicates that physical activity levels during recess are lower in older than in younger youths, particularly in girls [10-13]. In addition, levels of physical activity can be affected by the social context [14] and support [12], physical activity environment [12], and local school policies [15]. However, physical activity levels during recess may not be related to obesity [10,11,16]. Thus, in order to develop physical activity interventions, modifiable factors that are affecting physical activity levels during the recess period need to be understood.

Several instruments can be used to assess physical activity levels in adolescents [9]. Activity recalls can be answered quickly and are relatively easy to administer; however, such instruments are subject to a predefined activity list and recall error [17]. In contrast, direct observations, such as those obtained using the System for Observing Fitness Instruction Time (SOFIT), have the advantage of evaluating contextual information, including physical activity patterns and types, with the disadvantage of potentially misclassifying sedentary, light, and moderate intensity physical activities [18]. Finally, objective measures obtained using activity monitors, such as accelerometers, inform the actual time spent moving at different intensities; however, these activity monitors cannot identify the context of the physical activity.

Our understanding of physical activity levels can change depending upon the instrument being used to measure this behavior. The instrument could also influence the observed association between physical activity and health outcomes. Because a single instrument cannot fully capture physical activity patterns, it has been suggested that a combination of instruments be used [19].

The objectives of this study were to describe the physical activity patterns during school recess in a sample of 13–14 year old Mexican girls, and to examine differences in these patters as assessed using three physical activity measurement instruments. In order to better understand the influence of body weight status on the observed associations, analyses were also stratified by body mass index category.

## Methods

### Participants and procedure

Eighty-three female adolescents (13–15 years old) were recruited from the first to third years of secondary education at a private school in Mexico City. Nine students who initially volunteered withdrew from the study due to physical injuries, and two presented an abnormal resting electrocardiogram, leaving a sample size of 72. In an initial visit, participants attended the school clinic to undertake anthropometric and physical fitness measures, which were obtained by two trained observers. In a second visit, participants answered a general health survey and validated physical activity and behavioral questionnaires [20]. At the second visit we also measured physical activity during one recess period occurring in the main yard of the school using the SOFIT instrument, a physical activity recall, and Actical accelerometers (Mini Mitter Company, Bend, OR, USA). One week after the second visit, participants were instructed to wear an Actical accelerometer for 7 consecutive days, except when bathing or participating in water activities. In both the recess and 7-day measurement periods, the accelerometers were worn on the right hip and were programmed to collect data in 1-minute intervals (epochs).

### Recess context

The main yard or outdoor play area at the school was confined to basketball and volleyball courts. Students were not allowed to use balls, run, or scream unless they were on the courts. Recess was not directly supervised by teachers and principals. Recess duration was defined from when the bell rang to start recess at 11:00 am to when the bell rang to finish recess at 11:30 am. In this school, it was mandatory for every student to stay outside during recess. The 11:00–11:30 am period was the only daily recess break provided at this school.

### Anthropometry

Height and weight were measured using a portable stadiometer (SECA 220) and a digital scale (SECA 872). The body mass index (BMI, kg/m^2^) was calculated and overweight and obesity were determined using the International Obesity Task Force cut-points [21]. Waist circumference was measured three times using a fiberglass tape measure (Gülick) to the nearest 0.1 cm at the narrowest point between lower border of the rib cage and iliac crest. Average waist circumference was used for further analyses.

### Instruments

#### SOFIT

SOFIT can be used to quantify physical activity levels and the lesson context during recess and physical education classes. This method has been previously validated and the methodology is described elsewhere [18,22,23]. Trained observers recorded levels of physical activity during recess as lying down, sitting, standing, walking, or very active using a time sampling system of 10-second intervals. For the purpose of this study, lesson context observation and physical activity education classes were not evaluated.

Physical activity intensities were classified as follows: lying down and sitting as sedentary, standing as light physical activity, walking as moderate physical activity, and very active as vigorous physical activity [23]. Physical activity levels during recess were collected in groups of 4 participants within the same time interval (16 minutes); however, individual intervals were used for the purpose of the analysis. A total of 70% of the cases had valid SOFIT values. On average, two 10-second SOFIT intervals were collected for each participant.

#### Physical activity recall

The physical activity recall used in this study was developed by the research team. This instrument began by listing 20 frequently performed activities during the recess period. These consisted of 1) walking, 2) climbing down stairs, 3) looking for friends, 4) standing, 5) going to the store (eg, canteen or tuck shop), 6) sitting, 7) standing in line at the store, 8) talking, 9) hanging out with teachers, 10) lining up to go in and out of the classroom, 11) breaking line, 12) being punished, 13) going to the bathroom to look at the mirror, 14) going to the bathroom, 15) going to the flagpole, 16) walking up stairs, 17) going to the bathroom to wash hands, 18) skipping or jumping, 19) playing sports, and 20) running.

Immediately after the recess period concluded, participants were asked to recall which of the 20 activities they performed during the recess period. This was done separately for each 5-minute interval of the recess period. We assigned a metabolic equivalent (MET) to each activity according to the compendium of energy expenditures for youth [24] and then multiplied the MET value by the length of time spent at that level (5 min). Minutes spent in sedentary (≤1.5 METs), light physical activity (1.5 to 2.9 METs), moderate physical activity (3.0 to 5.9 METs), and vigorous physical activity (≥6 METs) were summed to obtain the total time spent during recess. The list of 20 physical activities were categorized as follows for the METs assignment: walking (1, 3, 5, 9, 11, 13–15, 17), sitting/standing talking (4,6,8), standing quietly (7,10,12), climbing stairs (2,16), skipping/jumping (18), playing sport games (19), or running/jogging (20). Results from this instrument are significantly correlated (p < 0.05) with accelerometer values for sedentary (r = 0.25), light (r = 0.29), and moderate-to-vigorous (r = 0.25) intensities.

#### Actical accelerometer

The Actical® is an omni-directional sensor that measures physical activity by accelerations in multiple directions, with more sensitivity in the vertical plane. The Actical detects accelerations in the range of 0.35-3.5Hz and g-forces of 0.05-2G, and has been tested and validated in adolescents [25]. Accelerometer data were downloaded and inspected using the manufacturer’s software (Actical V2.12, Mini Mitter Co. Bend, OR).

Different data management and cleaning procedures were used for the recess and weekly accelerometer data. For recess accelerometer data, the manufacturer’s software and the IBM SPSS Statistics software, version 20 (SPSS Inc., an IBM company Chicago Illinois, United States) were used to select the recess time period. Established cut-points were used for each epoch to determine whether the participant was sedentary (≤1.5 METs, <100 accelerometer counts) or engaged in light (1.5 to 2.9 METs, 100 to <1500 accelerometer counts), moderate (3.0 to 5.9 METs, 1500 to <6500 accelerometer counts), or vigorous (≥6 METs, ≥6500 accelerometer counts) intensity activity [26]. The number of minutes within each of the physical activity intensities was added to obtain the totals for each intensity category over the recess period. In addition to the previously mentioned procedures, weekly accelerometer data were cleaned using the Personal Activity and Location Measurement System (University of California, San Diego, California, United States). The same epoch criteria were used to determine physical activity intensity classification. Moreover, compliance criteria for wearing accelerometers were defined as a minimum of 4 weekdays of wearing the accelerometer for at least 10 hours per day [26]. All the periods of 60 or more consecutives minutes with zero epochs were removed prior to calculating wear time for a given day. Sleeping time during week and weekends reported in the behavioral questionnaire was used to remove sleeping time from the accelerometer data before the analysis. All of the participants had valid accelerometer data and therefore none were excluded from the analyses.

All participants and parents provided their written informed consent prior to participating. The National Public Health Institute Ethics Review Board of Mexico approved the study.

### Data analysis

Descriptive analysis was used to characterize participant characteristics and physical activity levels. Normality tests were calculated for all physical activity variables with Kolmogorov-Smirnov test. The mean proportion of time spent in the different physical activity intensities during the recess period and day was estimated. For the recess period, one-way ANOVA was used to compare activity intensity means between Actical, SOFIT, and the physical activity recall. The p value used to denote statistical significance was adjusted for multiple comparisons using Bonferroni’s method. For comparisons between body mass index categories, an independent *t*-test was used. The level of statistical significance was set at p < 0.05.

## Results

The demographic and anthropometric measures of the 72 female participants are presented in Table 1. The mean BMI was 21.4 (SD ± 3.7), 2.8% of the participants were underweight, 69.4% were normal weight, 25% were overweight, and 2.8% were obese.

**Table 1:** **Descriptive characteristics of adolescent participants, Mexico City, 2010 (N = 72) **

**Variable**	**Mean**	**Range**
Age (y)	13.5 ± 0.5	13.0 – 15.0
Height (cm)	158.6 ± 4.8	149.0 – 170.0
Weight (kg)	53.9 ± 9.6	38.5 – 78.2
Waist (cm)	69.8 ± 7.2	59.4 – 85.9
BMI (kg.m^−2^)	21.4 ± 3.7	15.7 – 32.2

**Figure 1:**
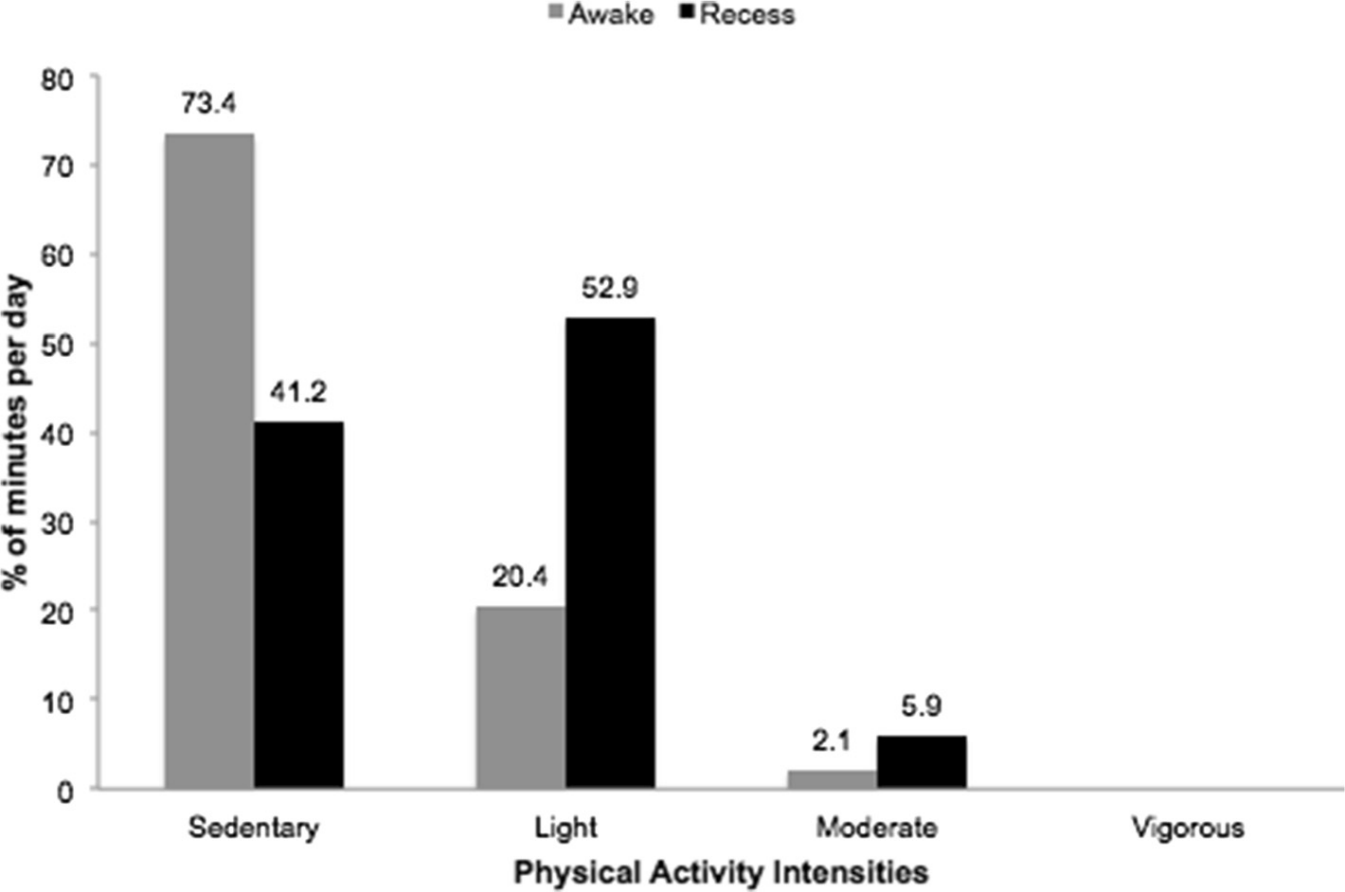
**Percentage of time spent awake and in the recess period categorized by intensity (average accelerometer counts per minute).** Mexico City, 2010.

Based on the weekly accelerometer data, the average recess time was 31 ± 2 minutes and represented 3.4% of the total waking time (≈15 hours). The percentage of time engaged in sedentary, light, moderate and vigorous physical activities during recess time at school account for 1.5%, 2%, 0.2% and 0% respectively of the total waking time. Based on the detailed accelerometer data assessment performed for one recess period, the proportion of the recess period spent at different intensities was 41.2% for sedentary behavior, 52.9% for light intensity, 5.9% for moderate intensity, and 0% for vigorous intensity, as shown in Figure 1. The corresponding values for waking time based on total time awake are also shown in Figure 1.

**Figure 2:**
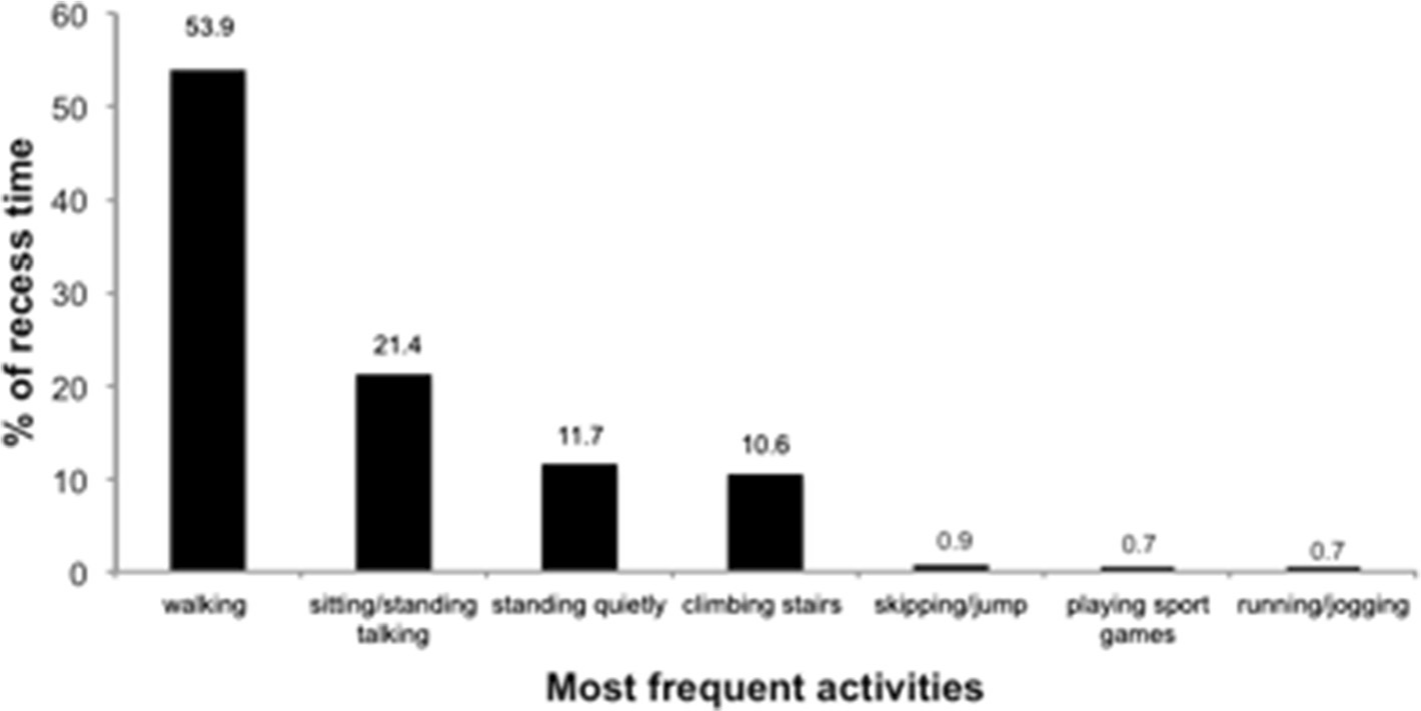
**Percent of recess period spent in different activities according to the physical activity recall.** Mexico City, 2010.

Figure 2 illustrates the proportion of the recess period spent in different activities according to the physical activity recall. Walking, talking while sitting or standing, and standing quietly were the most common activities. According to SOFIT, the percentage of the recess period spent in different activities was 54% for standing, 32% for walking, 13% for sitting, 1.7% for vigorous activities, and 0% for lying down.

The mean minutes of the recess physical activity performed at the different intensities by the three instruments is provided in Table 2. Significant differences were found across the instruments for all physical activity intensities. The same pattern was observed when stratified by weight status, with the exception of light and vigorous intensity activities in the overweight/obese group, where no statistical differences were found across instruments.

**Table 2:** **Average minutes of recess activity at various intensities using three different instruments, Mexico City, 2010**

		**Physical activity instrument**			
		**Accelerometer** ^**a**^	**Sofit** ^**b**^	**Recall** ^**c**^	**P value**
	**Physical activity intensity**				
*Total sample*	Sedentary	13.6 ± 5.8^b,c^	4.1 ± 7.0	5.2 ± 5.4	0.01
	Light	18.1 ± 5.3	17.0 ± 8.0^c^	20.7 ± 5.8	0.01
	Moderate	1.8 ± 2.1^b,c^	9.8 ± 5.9^c^	3.4 ± 3.2	0.01
	Vigorous	0 ± 0.1^c^	0.4 ± 0.9	0.7 ± 2.4	0.03
*Normal weight*	Sedentary	12.3 ± 5.7^b,c^*	3.9 ± 6.9	4.7 ± 4.5	0.01
	Light	19.3 ± 5.3*	16.9 ± 7.8^c^	21.1 ± 5.1	0.03
	Moderate	2.1 ± 2.3^b^	10.1 ± 6.3^c^	3.3 ± 3.3	0.01
	Vigorous	0 ± 0.1^c^	0.4 ± 0.9	0.9 ± 2.8	0.04
*Overweight/obese*	Sedentary	17.0 ± 4.9^b,c^*	4.8 ± 7.2	6.5 ± 7.1	0.01
	Light	15.3 ± 4.6*	17.1 ± 9.0	19.8 ± 7.2	0.13
	Moderate	1.0 ± 1.3^b^	8.8 ± 4.8^c^	3.5 ± 3.3	0.01
	Vigorous	0 ± 0.1	0.5 ± 1.1	0.3 ± 1.1	0.25

^a,b.c^Different super indices represent statistically significant differences between methods (p < 0.05).

*Statistically significant different between normal and overweight/obese groups (p <0.05).

Participants spent a significantly (p < 0.05) greater number of minutes in sedentary behavior based on accelerometers (13.6 ± 5.8) compared to the recall (5.2 ± 5.4) and SOFIT (4.1 ± 7.0). Overall, the overweight/obese group reported higher sedentary time compared to the normal weight group. In contrast, the number of minutes spent in light physical activity was not significantly different based on accelerometers and recall. Differences among other intensities are shown in Table 2.

Based on accelerometer data, the overweight/obese group reported significantly higher sedentary and lower light intensity minutes (17 ± 4.9 and 15.3 ± 4.6 respectively) compared to normal weight group (12.3 ± 5.7 and 19.3 ± 5.3 respectively) (p < 0.05). There were no significant differences between these BMI groups based on SOFIT and the physical activity recall data (Table 2).

## Discussion

The primary aim of this study was to describe the recess physical activity patterns in a sample of 13–14 year old Mexican girls. A secondary aim was to examine differences in the recess physical activity as captured using three different instruments. The third aim was to understand the influence of body weight status on the observed associations. Key findings are that the recess represents 3.4% of the daily waking time. The vast majority (94.1%) of the activities performed by adolescents during this period were of a light or sedentary intensity. Moreover, significant differences in the time spent in each intensity were found across the three physical activity instruments. Finally, based on accelerometer data, participants classified as overweight/obese had significantly higher sedentary minutes.

Based on the accelerometer data, we found that most of the recess time was spent in light and/or sedentary intensity activities. This result was consistent with the most common activities (standing and walking) reported by SOFIT and the physical activity recall and with what other authors have reported for Mexican children [23]. However, there were significant differences between minutes reported by the three instruments for all intensities, irrespective of BMI. Some factors that could potentially explain these differences include the fact that each instrument measures different physical activity parameters, potential misclassification of physical activities by interviewers, and the fact that only two-10 second SOFIT intervals were collected for each individual. This SOFIT interval may not accurately represent the activities children engage in during recess.

Based on accelerometer data, those participants classified as overweight/obese had significantly higher sedentary and lower light intensity minutes compared to normal weight participants. Martinez-Gomez et al. [11] found no significant associations between BMI and MVPA levels during recess among Spanish adolescents. In addition, Hohepa et al. [16] found that New Zealand adolescents classified as overweight/obese were 27% more likely to be in the “more active group” (mostly of the time played active games) during the morning recess than were non-overweight/obese adolescents. Differences in the results could be related to the fact that both studies used a self-reported physical activity measure. Indeed, in our study no significant differences were found across BMI groups for the self-reported physical activity recall.

According to global physical activity recommendations, adolescents should accumulate at least 60 minutes of MVPA on a daily basis [27]. Accelerometer data from this study suggest that these adolescent girls only spent 19 minutes per day, on average, engaged in MVPA. Thus, adolescents from this study only accumulated one third of the amount of recommended MVPA [27] and less physical activity than what others have reported internationally for this age group [26,28].

Some authors have suggested that the time spent during recess may contribute between 5 and 40% of the daily physical activity recommendation [8]. Results from this study indicate that the volume of MVPA accumulated during the recess period only contributed to 3.3% (approximately 2 minutes) of the daily recommendation. This is less than what Bailey et al. [29] found for 10–14 year old girls from the UK. That study reported that girls accumulated an average of 5.3 minutes and 15.3 minutes of MVPA in a 15–20 min morning recess and 45–65 min lunch recess, respectively. Another study performed by Martinez-Gomez et al. [30] reported that 10–14 year old Spanish girls spent 3.9 minutes in moderate physical activity and 8.7 minutes in vigorous physical activity according to accelerometer data, and 7.6 minutes in moderate and 3.5 minutes in vigorous physical activity according to recall data in a 25-min recess period. In another study of 3471 participants (mean age 14 years) from New Zealand, 19.9% and 14.3% of the adolescents were involved in “more active” (mostly of the time played active games) lunchtime and morning recess respectively [16]. Finally, another study reported lower physical activity minutes than our study. He et al. [13] found that 13 year old Japanese girls spent on average 0.6 minutes of physical activity during approximately 45 minutes of lunchtime.

Among the reasons that can explain the short period of time spent in MVPA during recess include the high percentage of time spent in school policy-related activities such as lining up to go in and out of the classroom, walking up and down stairs, and punishments (eg, when a student misbehaves they are often punished by being asked to stay inside the classroom or stand beside the teacher during recess). In addition, a significant proportion of the recess period can be spent in food-related activities such as standing in line at the school food store and/or eating food. This is particularly relevant in Mexico since the recess period is the only time in which children can eat food during the school day.

Another reason for the low physical activity levels may relate to the girls’ social context. Results from some studies indicate that boys are more likely to get involved in ball-based games and girls in sedentary activities such as socializing with friends, standing, and looking for friends [14,31,32]. Finally, some authors have found that the low levels of physical activity during the recess are related to reduced playground spaces, physical activity restriction, and the lack of sports equipment [8,15,33]. Although this was a two-floor school with basketball and volleyball courts, students were not allowed to use balls or run outside of the court area during recess.

Other authors have proposed a variety of approaches to increase physical activity levels during recess. Zask et al. [34] suggested that the length of recess be increased to augment physical activity levels. Haug et al. [15] stipulated that schools with organized physical activities during non-curricular school time and a written policy for physical activity had a higher proportion of students reporting physical activity during recess. Verstraete et al. [35] and Haapala et al. [36] found that providing game equipment during recess time increased physical activity levels in children. Haapala et al. [36] also reported that accounting for individualization and gender-sensitivities increased physical activity levels within the female group. In two systematic reviews Ickes et al. [37] and Parrish et al. [38] found that playground marking, playground zones, teacher involvement, and active video games are also successful strategies to increase physical activity levels. Finally, in Mexican schools it may be beneficial to separate the single 30 minutes recess period in two 15-minute recess periods, one focused on eating and the other on physical activity.

There are some limitations of this study that need to be addressed. Physical activity levels differ depending on the accelerometer cut-points used. When lower cut-points were selected, smaller and non-significant differences were found between the accelerometer and SOFIT (data not shown). In addition, a threshold of 3 METs was used to define moderate intensity physical activity, and there has been some debate in the literature as to whether a threshold of 4 METs may be more appropriate [39]. In addition, the study sample was chosen by convenience and the use of a single school of 72 girls limits the generalizability of the findings.

Another potential limitation in our study was the lack of intra-individual variation information within the physical activity methods. Although attempts were made to estimate accelerometer intra-individual variation using the 7-day data collection and extracting the theoretical recess time (weekdays 11:00–11:30 am), these estimations were not comparable to our one-recess accelerometer data since recess time was not verified (i.e. absenteeism during recess and unrelated activities performed during recess time, such as festivals or sport tournaments, were not recorded). Finally, future studies should include a sample of males to allow for gender comparisons.

## Conclusions

In conclusion, female adolescents aged 13–14 years spent 94.1% of their total recess time performing light intensity or sedentary activities. Future studies within Mexico should consider developing interventions to increase physical activity levels during recess.
